# Subcutaneous emphysema during surgical gingival depigmentation: a case report

**DOI:** 10.1186/s13256-024-04711-z

**Published:** 2024-10-22

**Authors:** Rasha Attia, Neveen Nabil, Maged Anis

**Affiliations:** 1 Department of Oral Medicine and Periodontology, Faculty of Dentistry, October University For Modern Sciences and Arts (MSA Uni), Giza, Egypt; 2 Wahat Road, Giza, Egypt

**Keywords:** Emphysema, Gingival depigmentation, Dental complication, Crepitus, Diagnosis

## Abstract

**Background:**

Subcutaneous emphysema related to dental procedures is well documented in literature. It usually occurs during or within minutes to hours after dental treatment and can be easily diagnosed by the presence of crepitus on palpation. Although it is self-limiting, it can develop to potentially life-threatening complications.

**Case presentation:**

To the authors knowledge, this is the first report documenting the development of subcutaneous emphysema in a 22-year-old Egyptian female during lower surgical gingival depigmentation using NSK high speed, air driven handpiece operated at 0.25 MPa. Sudden swelling developed involving the right side of the face and immediate diagnosis of subcutaneous emphysema was made on the basis of the presence of crepitus during palpation of the swollen area. Intraoral examination revealed small tissue laceration of the loosely attached alveolar mucosa through which the pressurized air might have passed into the fascial spaces. Complete resolution of the swelling occurred after 7 days without further complications.

**Conclusion:**

Straightforward surgical procedures, such as gingival depigmentation, can be complicated by the development of subcutaneous emphysema. The crucial role of dentists is to be aware of its signs and to immediately diagnose and manage it to avoid further complication.

## Background

Subcutaneous emphysema (SE) is rare but serious clinical complication that may occur during or after dental procedures [1–3]. It occurs owing to forceful injection of air into the loose surrounding connective tissue [4]. Clinically, it appears as swelling with crepitus on palpation, and may spread along the fascial planes [4, 5]. It can lead to soft tissue infection, air embolism, pneumothorax, and some cases are considered life-threatening if not diagnosed early and properly treated [4, 6–8].

The fascial planes of the head and neck consist of loose connective tissue and once the pressured air enters the soft tissue, it will follow the path along the fascial planes, spreading to distant spaces. Air passes through the neck from the submandibular space to enter the retropharyngeal space, which lies between the posterior wall of the pharynx and the vertebral column. In some cases, the air may reach the posterior mediastinum where it can compress the venous trunks resulting in cardiac failure, or compress the trachea resulting in asphyxiation.

Treatment of subcutaneous emphysema is based on the severity of the condition and the clinician experience. In mild cases, resolution begins after 2–3 days with complete resolution occurring after 7 days. Close monitoring of the patient and reassurance are enough. In case of significant discomfort, microdrainage using subcutaneous fenestrated catheter inserted in the area of the emphysema with sequential massage could be considered. Clinicians prefer antibiotic administration as a prophylaxis against secondary infection and when emphysema occurred after managing infected tooth, especially in case of cervicofacial emphysema as it may lead to deep neck infections [9]. In severe life-threatening cases, immediate medical attention is needed, and securing the airway has the highest priority [10].

The use of high-speed air handpieces is considered the most common risk factor for development of SE, especially during surgical tooth removal [1, 2]. Gingival hyperpigmentation has a major negative effect on esthetics. Various surgical and non-surgical techniques were developed to eliminate or reduce this hyperpigmentation, including blade surgery, bur abrasion, cryotherapy, electrosurgery, free gingival autografting, soft tissue allografts, lasers, and chemical peeling methods. The current report describes an unusual clinical case of SE that occurred during surgical bur abrasion depigmentation of the lower gingiva using a high speed handpiece.

Our goal is to share this rare clinical experience with the dental clinical practitioners to raise awareness of the clinical causes of iatrogenic SE that may complicate simple dental treatment. Additionally, to highlight the importance of proper immediate diagnosis of these clinical manifestations.

## Case report

**Fig. 1:**
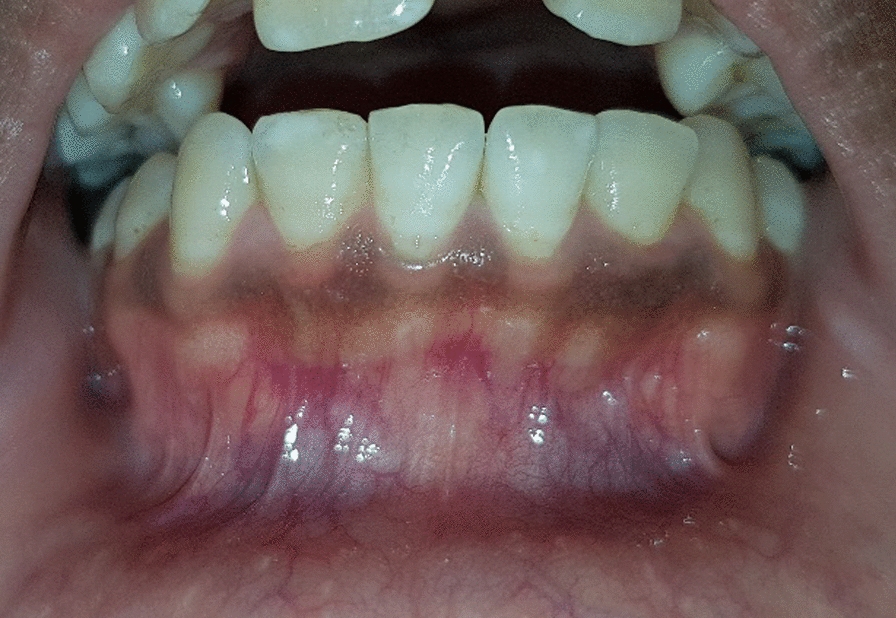
Preoperative clinical photo of the gingival pigmentation

A 22-year-old Egyptian female presented to MSA University Periodontology clinic for gingival depigmentation of the lower arch. The intraoral examination showed brown pigmentation of the lower gingiva that was classified according to the melanin index as class 2 and score 3 according to oral pigmentation index (Fig. 1). Periodontal probing showed sulcular depths of 1–2 mm with no bleeding on probing. The patient reported normal medical history with no history of smoking or previous drug allergies.

The treatment options were discussed with the patient, and surgical bur abrasion technique was the treatment of choice. To ensure that the patient fully comprehended the procedure, the benefits, and the risks related to any surgical complications and their treatment, she was asked to voluntarily sign an informed consent before the surgery.

A high speed handpiece operated at 0.25 MPa with friction grip football shaped medium grit diamond bur and copious irrigation were used to execute the surgical procedure. Under local anesthesia, brushing motions with minimal pressure were applied by the abrasive bur to remove the pigmented epithelium with a thin layer of the underlying connective tissue. The exact deepithelization depth cannot be determined; however, brushing motions were stopped once pinpoint bleeding was noticed. The denuded surgical area was irrigated with saline and sterile gauze was used with light pressure to control the bleeding. Care was taken to avoid holding the bur in one place during the brushing strokes to prevent tissue damage.

During the surgical procedure on the right side, the patient felt sudden severe pain and developed acute swelling on the right side of her face. The surgical procedure was interrupted, and the operator tried to reassure the patient. The patient was alert and responsive but confused and showed normal vital signs without any signs of respiratory distress.

**Fig. 2:**
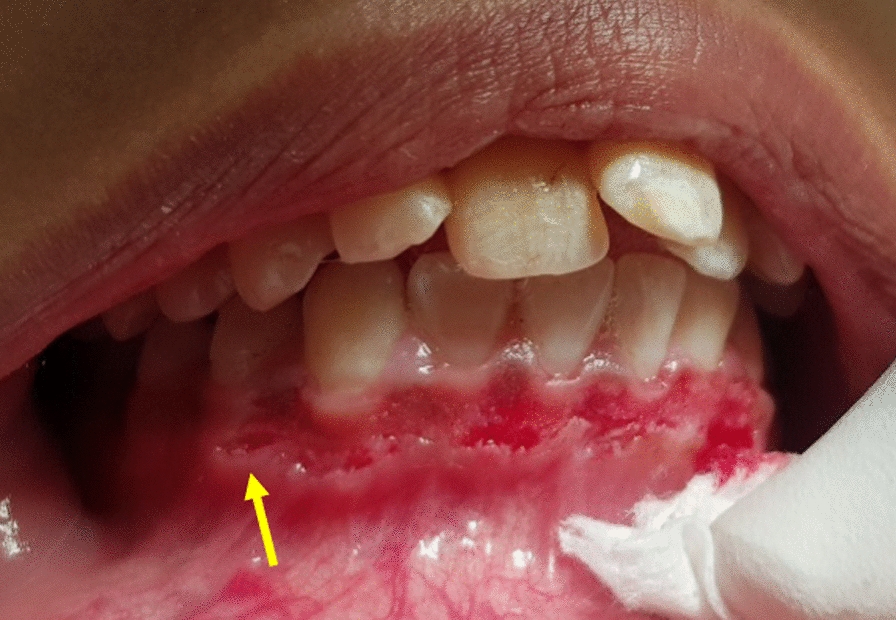
Clinical presentation of the tissue laceration between the lower right canine and first premolar (yellow arrow)

Intra- and extraoral examinations were performed. The extraoral examination revealed unilateral swelling of the right side of the face extending from the upper eyelid region to the submandibular region. The right eye was swollen and closed, but with intact visual acuity and ocular movement. Upon palpation, crepitus was evident involving the area from the temporal part of the face, the upper eyelid, the right side of the face, beyond the right ear, and the right side the neck. The patient reported no pain and tenderness was not detected during extraoral palpation. Intraoral examination revealed a small soft tissue laceration of approximately 4 mm mesiodistally at the mucogingival junction involving the alveolar mucosa between the right lower canine and the first premolar (Fig. 2).

Subcutaneous emphysema was the definitive clinical diagnosis based on the presence of crepitus. The patient was reexamined by the university physician to confirm the diagnosis, monitor her condition, and to discuss the treatment approach. The patient was reassured and monitored in-office for 2 hours and, since she was asymptomatic and was able to open her eye, she was discharged. Amoxicillin at 500 mg was prescribed for 7 days as prophylactic therapy to prevent bacterial infection.

She was instructed to avoid any activities that would increase intraoral air pressure e.g., straw drinking, oral blowing, and sneezing. She was also advised to adhere to the follow-up schedule to monitor the condition. The patient was contacted by phone the night of the incident and she reported slight resolution of the swelling. The patient was reexamined on the second, fourth, and seventh post-emphysema development days. Complete resolution of the swelling and crepitus were noted after 7 days without further complications and with uneventful healing of the intraoral tissue laceration. The patient was followed up for 2 months to monitor any long-term complications related to SE.

## Discussion

The current case report described the diagnosis, clinical signs, and management of SE that occurred during surgical gingival depigmentation using abrasive stone with air-driven high-speed handpiece. SE is a clinical probability, especially with the use of air-driven handpieces, and it can interrupt and complicate a minor straightforward surgical procedure, such as gingival depigmentation, and can even lead to life-threatening condition if not immediately diagnosed and managed.

Despite being considered a benign intraoral condition, gingival hyperpigmentation is also considered an esthetic problem especially in individuals with gummy smile. Excessive gingival pigmentation is frequently caused by deposition of the brown melanin pigment in the suprabasal layers by the active melanocytes of the gingival epithelium [11, 12].

Gingival depigmentation is one of the periodontal plastic surgeries aiming to remove or reduce gingival hyperpigmentation by removal of the superficial epithelial layers using various modalities [13]. Treatment modalities were classified into surgical and non-surgical techniques. Surgical methods include (1) techniques that aim to remove hyperpigmentation, such as scalpel surgical technique, bur abrasion method, electro-surgery, cryosurgery, radiosurgery, and lasers, and (2) techniques that are used to mask the hyperpigmentation, such as free gingival graft and acellular dermal matrix allograft. The nonsurgical technique includes chemical peeling of the gingival pigments using different agents, such as phenols, alcohol, and ascorbic acid [13, 14].

There is no ideal technique for gingival depigmentation as each technique has its own advantages and disadvantages and the choice of one technique over the other is based on the clinician’s skills, the available equipment, the procedure cost, and patient preference [15].

Being the most economic technique as it does not require extensive equipment, and attributed to faster healing in comparison with other techniques, the scalpel method causes bleeding during and after the procedure and is known for a high repigmentation rate [16].

Electrosurgery on the other hand, controls the bleeding, causes less patient discomfort with minimal scar formation and shorter chair time. Despite its advantages, electrosurgery requires more skillful operators to avoid undesired soft and hard tissue destruction. Radiosurgery is an advanced form of electrosurgery in which electromagnetic energy is used for soft tissue removal. Radiosurgery produces coagulation in the surgical area, which controls the bleeding, and it also provides faster healing in comparison with scalpel and laser therapy, but the complete therapy requires at least two sessions over 2 weeks [13].

Cryosurgery has the advantages of easy application, no hemorrhage, no scar formation, and is performed without anesthesia. However, it may be accompanied by considerable swelling and tissue destruction may occur owing to prolonged freezing time [13]. Laser therapy provides visible dry bloodless surgical field during gingival depigmentation. Although it is considered an effective and reliable technique, laser disadvantages include prolonged wound healing, thermal tissue damage, and the high costs [15, 17].

Chemical peeling using phenol and alcohol was considered a harmful method, which may leads to soft tissue necrosis and pain. Ascorbic acid has been used topically with dermapen and yielded promising results regarding depigmentation of the gingiva. This technique should be used by skillful clinicians to avoid tissue destruction and gingival recession [18].

Hyperpigmented gingival tissues can be replaced by unpigmented free gingival autograft. Second surgical site, increased patient discomfort, and tissue color mismatch are the major disadvantages of this invasive technique. On the contrary, acellular dermal matrix allograft is less invasive and is more efficient in providing esthetically accepted gingival tissues by eliminating or reducing the gingival pigmentation. However, it is considered more invasive than other surgical depigmentation techniques [17].

Bur abrasion technique is one of the surgical techniques developed for surgical gingival depigmentation. It is relatively safe and simple and is considered affordable with short chair time. This technique requires only an abrasive stone and air-driven handpiece, which are available in every dental clinic [19].

On the basis of the authors’ literature search, the reported complications related to gingival depigmentation procedures were postoperative pain, soreness, bleeding, and tissue damage or necrosis, but SE development in relation gingival depigmentation was not reported.

The first documented dental SE was more than 100 years ago by Turnbull after extraction of bicuspid tooth [20]. Although it is considered a rare condition, a recent systematic review showed a significant increase in frequency of dental SE [1].

The etiology of SE is either patient-related or iatrogenic. SE can be induced by the patient coughing, forceful blowing, smoking, or vomiting after the dental procedure [2]. The most common iatrogenic factor reported in literature was use of the air-driven dental handpiece [1, 2, 21]. In their systematic review, Jones *et al*. reported that 51.1% of the SE cases resulted from the use of an air-driven dental handpiece with 62% of these cases occurring following surgical tooth removal, 28% after restorative procedure, such as a restoration or crown preparation, and 10% occurred after nonsurgical endodontic procedures. The use of air-syringe, air-polishing/prophylaxis systems, and dental lasers was reported to induce SE [1, 22–26]. Moreover, cases of SE have been reported in association with endotracheal intubation and positive pressure ventilation [1, 2]. In the current case, SE occurred owing to the use of the air-driven handpiece, although the air entry access to the fascial planes was uncertain. It was assumed that the forceful air entered through either the gingival sulcus, which was of normal depth, or through the small laceration in the alveolar mucosa. Overall, 60% of SE occurs during or after dental procedures in the mandible, especially the posterior segment. Explanation is related to the anatomical structure of this region that possibly allows easier path for the forceful air to enter and spread by dissecting the tissues [1].

With the use of high-speed handpiece, the air could forcefully pass into the fascial spaces of the face and neck. This may cause facial emphysema if the forceful air gets localized in the face or cervicofacial emphysema if it spreads to the neck and mediastinal, pleural, or peritoneal emphysema when it descends to involve the thorax or abdomen [27]. The pressurized air may be forced into the soft tissue through the root canal system, gingival sulcus, especially in patients with deep periodontal probing depths, or tissue lacerations [23, 28, 29].

In the present case, the periodontal probing depth of the patient was 1–2 mm with normal gingival tissue, so the gingival sulcus was not likely to be the point of air entry. However, soft tissue laceration that occurred in the alveolar mucosa mesial to the right first premolar was believed to be the point through which the compressed air passed into the fascial spaces. Once the air penetrated the soft tissues, it dissected the fascia and passed to submandibular space, which is directly communicated with lateral pharyngeal space. The air then passed to the retropharyngeal space and accumulated where the fascia attaches to the clavicle.

Goodnight *et al*., stated that the most important immediate step in the management of SE is the correct diagnosis [4]. Clinically, SE presents as rapid onset soft tissue swelling with crepitus on palpation of the involved tissue and lack of significant tenderness, erythema, or lymph node involvement [1, 4, 29]. Dysphonia, dysphagia, and dyspnoea may be present [1]. Pain can be associated with SE owing to tension in the involved tissues [24]. The diagnosis can also be confirmed by the presence of air in the X ray of the affected area [10, 22, 23].

Crepitus on palpation is considered the pathognomonic sign of SE that differentiates it from other clinical causes of facial swelling, such as anaphylaxis, angioedema, hematoma, mucocele, and facial cellulitis [1, 2, 28]. Crepitus or crackling is the bubbling sensation upon palpation of the swelling. In the current case, diagnosis of SE was immediately confirmed by the presence of the crepitus and lack of tender palpation of the affected area.

A recent systematic review reported that although most SE cases are self-limiting and begin to resolve spontaneously after 2–3 days, broad spectrum prophylactic antibiotics were prescribed in 76% of the cases [1]. The rationale for antibiotic prophylaxis is that air entering the tissues is contaminated with oral bacteria and other debris that could potentially lead to rapidly spreading cellulitis or necrotizing fasciitis [2, 28–30]. Analgesics are rarely prescribed as pain is often minimal [29]. Incision and drainage are not required for air removal as air is naturally absorbed and lost within soft tissue. It can also be an additional route for air inflow and bacterial contamination [30]. In this case, only Amoxicillin was prescribed to prevent any potential bacterial infection.

One important aspect in the treatment of SE is patient-centered management, which includes reassurance, monitoring, and education. The development of SE is one of the most stressful situations patients may experience in the dental office, and great efforts should be directed to reassure the patients to decrease their anxiety and to allow for better handling of the case.

Although it is rare for SE cases to progress into life-threatening conditions, such as pneumothorax, air embolism, and mediastinitis, close monitoring of the patient is necessary to allow rapid intervention to prevent or to treat any of these potential complications if they occur [10, 29, 30].

Patient education about SE is an integral part of the treatment plan. Patients should be educated about the nature and the course of this pathological condition. They should be instructed to avoid any behavior that may increase intraoral pressure, such as forced exhalation, coughing, smoking, and gargling. Additionally, they should avoid lying in supine position, which may worsen the condition [30]. Strict instructions to the patient to visit emergency room in case of increased swelling or difficulty breathing after being dismissed from the dental office.

## Conclusion

Raising the awareness of the possibility of SE development during or after dental procedures is mandatory among dental practitioners, especially with the use of air-driven handpieces. Straightforward surgical procedures, such as gingival depigmentation, can be complicated with the development of SE. The crucial role of the dentists is to be aware of its signs and to immediately diagnose and manage it to avoid further complications. Dentists should know when to consider referral of the patient to the emergency room for further investigations. Although bur abrasion surgical technique is a simple, low-cost, and effective method for gingival depigmentation, it implies high risk for developing SE owing to the use of air-driven handpiece. Clinicians should consider other safer techniques, such as blade surgery or chemical peeling with ascorbic acid, or if bur abrasion is the technique of choice, they should use an electric handpiece, which is powered by electricity rather than compressed air.

## Data Availability

Not applicable.

## Abbreviation

SE: Subcutaneous emphysema
